# Polygenic risk scores for coronary artery disease and subsequent event risk amongst established cases

**DOI:** 10.1093/hmg/ddaa052

**Published:** 2020-03-27

**Authors:** Laurence J Howe, Frank Dudbridge, Amand F Schmidt, Chris Finan, Spiros Denaxas, Folkert W Asselbergs, Aroon D Hingorani, Riyaz S Patel

**Affiliations:** 1 Institute of Cardiovascular Science, Faculty of Population Health Sciences, University College London, London NW1 2DA, UK; 2 Department of Health Sciences, University of Leicester, Leicester LE1 7RH, UK; 3 Department of Cardiology, University Medical Center Utrecht, University of Utrecht, Utrecht, CX 3584, The Netherlands

## Abstract

**Background:**

There is growing evidence that polygenic risk scores (PRSs) can identify individuals with elevated lifetime risk of coronary artery disease (CAD). Whether they can also be used to stratify the risk of subsequent events among those surviving a first CAD event remain uncertain, with possible biological differences between CAD onset and progression, and the potential for index event bias.

**Methods:**

Using two baseline subsamples of UK Biobank: prevalent CAD cases (*N* = 10 287) and individuals without CAD (*N* = 393 108), we evaluated associations between a CAD PRS and incident cardiovascular and fatal outcomes.

**Results:**

A 1 SD higher PRS was associated with an increased risk of incident myocardial infarction (MI) in participants without CAD (OR 1.33; 95% CI 1.29, 1.38), but the effect estimate was markedly attenuated in those with prevalent CAD (OR 1.15; 95% CI 1.06, 1.25) and heterogeneity *P* = 0.0012. Additionally, among prevalent CAD cases, we found an evidence of an inverse association between the CAD PRS and risk of all-cause death (OR 0.91; 95% CI 0.85, 0.98) compared with those without CAD (OR 1.01; 95% CI 0.99, 1.03) and heterogeneity *P* = 0.0041. A similar inverse association was found for ischaemic stroke [prevalent CAD (OR 0.78; 95% CI 0.67, 0.90); without CAD (OR 1.09; 95% CI 1.04, 1.15), heterogeneity *P* < 0.001].

**Conclusions:**

Bias induced by case stratification and survival into UK Biobank may distort the associations of PRS derived from case-control studies or populations initially free of disease. Differentiating between effects of possible biases and genuine biological heterogeneity is a major challenge in disease progression research.

## Introduction

Coronary artery disease (CAD) is heritable, with over 300 independent genetic loci with additive effects known to influence disease risk having been identified in Genome-Wide Association Studies (GWAS) (1–4). Exploiting the increasing amount of risk variation captured by identified loci, recent studies have illustrated the potential use of CAD polygenic risk scores (PRSs) for identifying individuals at an elevated risk of CAD (5–7), where the scores are based on the counts of the number of risk alleles carried.

**Figure 1:**
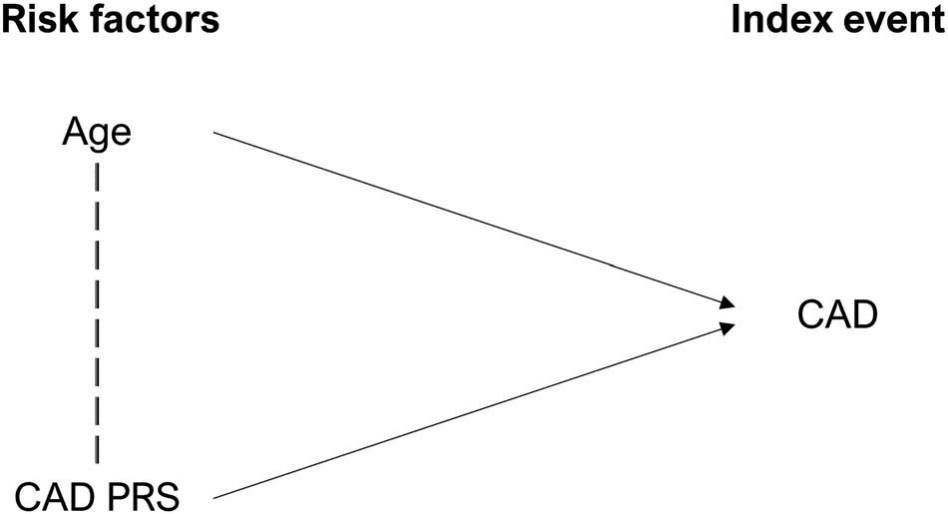
A directed acyclic graph displaying an index event CAD status, with two risk factors: increased age and CAD PRS. The dotted line between Age and CAD PRS indicates that when conditioning on the index event, associations are likely to be induced between the two risk factors. For example, if an individual develops CAD at the age of 20, this suggests that they are likely to have a high CAD PRS.

**Figure 2:**
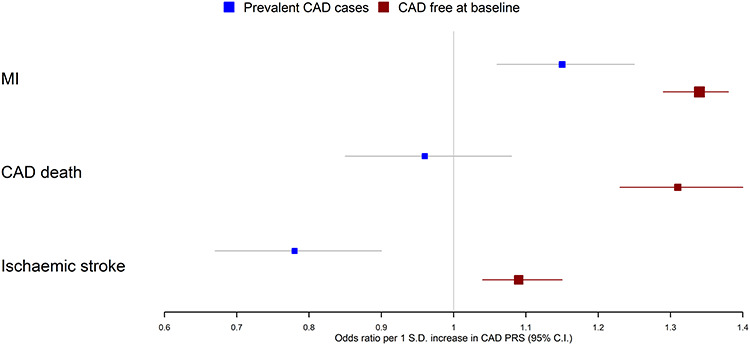
Associations between a CAD PRS and incident MI, CAD death and ischaemic stroke in prevalent CAD cases and in individuals free of CAD at baseline. Note that these three outcomes were chosen based on strength of evidence for heterogeneity between the case/CAD free individuals.

However, the extent to which CAD PRS derived from general population cohorts or case-control GWAS are associated with CAD disease progression, as characterized by subsequent or recurrent events amongst diseased cases, remains unclear. Indeed, established risk variants for onset of CAD may not necessarily equate with variants influencing risk of subsequent events because of genuine aetiological differences between the pathophysiology of the two states (8,9). Alternatively, even if variants influencing disease onset also genuinely influence progression, associations may be distorted because of index event bias, where conditioning on an index event (e.g. presence of CAD) may induce confounded associations between risk factors in the sample of individuals with the index event (Fig. 1) (10,11). The lack of a strong association between the major CAD risk locus at 9p21 and subsequent event risk highlights possible differences in the genetic associations of CAD risk variants dependent on case status (12,13).

Previous studies have found some evidence that CAD PRSs are associated with an increased risk of subsequent events [e.g. recurrent MI and revascularization (Revasc)] (7,14–19), although a recent study in a French–Canadian population found that CAD PRSs are substantially less effective at predicting recurrence and incident cases than prevalence (7). Stronger conclusions have been limited by the modest sample sizes of recurrence studies, with most previous studies including less than 5000 cases, as well as the inconsistency of cardiovascular (CV) and fatal endpoints across different studies.

Using two subsamples of UK Biobank, defined as (a) individuals free of CAD and (b) those with evidence of prevalent CAD at enrolment, we aimed to evaluate the extent to which associations between CAD genetic risk variants and incident events differ when restricting to a case-only sample, while also running several exploratory analyses to detect and account for potential index event bias.

## Results

### CAD PRS and incident events

Associations between the PRS and incident events differed greatly between the CAD free and prevalent CAD case samples, with 95% confidence intervals non-overlapping for 8 out of the 10 outcomes tested (heterogeneity *P* < 0.05). In the CAD-free sample, we found strong evidence of positive associations between the CAD PRS and incident CV and fatal outcomes such as MI (OR 1.34; 95% CI 1.29, 1.38), CAD death (OR 1.31; 95% CI 1.23, 1.40) and ischemic stroke (OR 1.09; 95% CI 1.04, 1.15). In contrast, in the prevalent CAD sample, we found evidence of a positive, but attenuated association with MI (OR 1.15; 95% CI 1.06, 1.25; Int *P* = 0.012), weak evidence for an association with CAD death (OR 0.96; 95% CI 0.85, 1.08; Int *P* = 9.1 × 10^−6^) and evidence of inverse associations with all-cause death (OR 0.91; 95% CI 0.85, 0.98; Int *P* = 0.0041) and ischemic stroke (OR 0.78; 95% CI 0.67, 0.90; Int *P* = 1.8 × 10^−5^) (Fig. 2; Table 1). Amongst prevalent CAD cases, we did not find strong evidence of heterogeneity by CAD subtype (CAD without prior MI, CAD with prior MI) with overlapping confidence intervals across all outcomes (Supplementary Material, Table S1).

**Table 1:** Associations between CAD PRS and incident events

Outcome	No CAD at baseline (*N* = 393 108) OR^a^ (95% CI)	Prevalent CAD cases (*N* = 10 287) OR^a^ (95% CI)	Heterogeneity *P*-value
CAD death/MI	1.33 (1.29, 1.38)	1.11 (1.03, 1.20)	1.4 × 10^−5^
CV death	1.20 (1.14, 1.26)	0.97 (0.87, 1.09)	0.0012
All-cause death	1.01 (0.99, 1.03)	0.91 (0.85, 0.98)	0.0041
CAD death	1.31 (1.23, 1.40)	0.96 (0.85, 1.08)	9.1 × 10^−6^
MI	1.34 (1.29, 1.38)	1.15 (1.06, 1.25)	0.0012
Revasc	0.99 (0.97, 1.01)	0.91 (0.84, 1.00)	0.067
Heart failure	1.00 (0.93, 1.08)	0.97 (0.86, 1.10)	0.67
Stroke	1.05 (1.01, 1.09)	0.88 (0.78, 1.00)	0.0094
Ischaemic stroke	1.09 (1.04, 1.15)	0.78 (0.67, 0.90)	1.8 × 10^−5^
All CVD	1.06 (1.04, 1.08)	0.99 (0.93, 1.04)	0.013

^a^All OR per 1 SD increase in CAD PRS of 182 SNPs.

### CAD PRS and baseline covariates

The CAD PRS was inversely associated with age, body mass index (BMI) and smoking initiation and positively associated with statin use in both samples, with some evidence of larger effect sizes in the CAD sample for age (Int *P* = 0.0064), statin use (Int *P* < 0.001) and BMI (Int *P* = 0.011). In contrast, we found some evidence that the CAD PRS is associated with increased systolic blood pressure (SBP) in those without CAD, but this association was largely attenuated in the prevalent CAD sample (Int *P* = 0.020). Similarly, the direction of effect estimates differed between the two samples for type II diabetes with some weak evidence of heterogeneity (heterogeneity *P* = 0.053) (Table 2; Supplementary Material, Figs S1–S7).

**Table 2:** Associations between CAD PRS and covariates

Covariate		Values of covariates at quintiles of the CAD PRS distribution				Heterogeneity *P*-value^a^
		20%	40%	60%	80%	
Age (years)	No CAD at baseline (*N* = 393 108)	66.7	66.7	66.6	66.6	0.0064
	Prevalent CAD cases (*N* = 10 287)	72.3	72.1	72.0	71.9	
Sex (male = 1, female = 0)	No CAD at baseline (*N* = 393 108)	0.47	0.45	0.44	0.43	0.84
	Prevalent CAD cases (*N* = 10 287)	0.82	0.80	0.79	0.77	
Statin use (Yes = 1 No = 0)	No CAD at baseline (*N* = 393 108)	0.04	0.10	0.16	0.24	2.3 × 10^−5^
	Prevalent CAD cases (*N* = 10 287)	0.67	0.81	0.93	>1.0	
Type II diabetes (Yes = 1 No = 0)	No CAD at baseline (*N* = 393 108)	0.03	0.04	0.05	0.05	0.053
	Prevalent CAD cases (*N* = 10 287)	0.21	0.19	0.17	0.14	
SBP (mmHg)	No CAD at baseline (*N* = 393 108)	139.4	139.7	139.9	140.2	0.020
	Prevalent CAD cases (*N* = 10 287)	139.3	139.4	139.4	139.4	
BMI (kg/m^2^)	No CAD at baseline (*N* = 393 108)	27.2	27.1	27.1	27.1	0.011
	Prevalent CAD cases (*N* = 10 287)	29.1	29.0	28.9	28.9	
Smoking (Ever = 1 Never = 0)	No CAD at baseline (*N* = 393 108)	0.45	0.44	0.44	0.44	0.11
	Prevalent CAD cases (*N* = 10 287)	0.70	0.67	0.65	0.63	

^a^Test for heterogeneity between regression estimates in prevalent case and control samples.

In the whole unstratified UK Biobank sample (*N* = 408 480), we found no strong evidence of an association between the CAD PRS and age or smoking but found some evidence that a higher PRS is associated with an increased risk of type II diabetes, increased SBP, reduced BMI and increased statin use (Supplementary Material, Table S2).

### Accounting for index event bias

**Figure 3:**
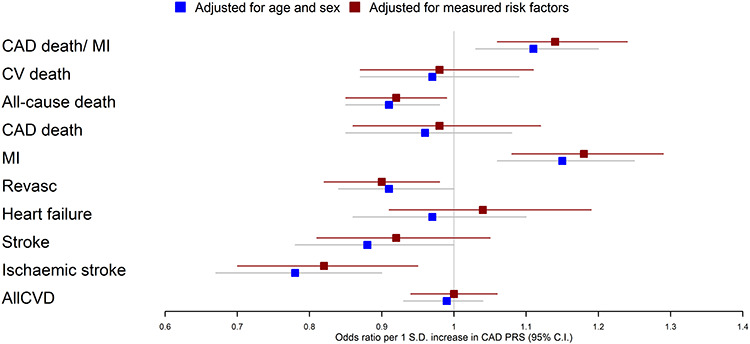
Associations between a CAD PRS and 10 incident fatal/CV outcomes. One analysis (blue) only included age and sex as covariates, while the other analysis (red) included additional CAD risk factors as covariates (BMI, SBP, statins, type II diabetes and ever smoking status).

First, we included CAD risk factors (SBP, BMI, smoking and diabetes) and statin use as covariates in the model to account for potential index event bias. Note that adjusting for covariates will not account for confounding relating to unmeasured covariates. Although estimates in general moved slightly closer to the non-case estimates from Table 1, we did not find an evidence of discernible statistical differences when including these covariates (Fig. 3; Supplementary Material, Table S3).

Second, we applied a method to correct for index event bias in GWAS (20). The regression of genetic effects for prognosis on those for incidence generated a positive slope estimate (a measure of index event bias) using both SIMEX (0.0655; 95% CI 0.646, 0.0664) and the Hedges-Olkin estimator (0.0516). Across the 5564 SNPs used, the *I*^2^ G_X_ statistic was 89.0%, consistent with some evidence of modest measurement error. Prior to the adjustment using the SIMEX estimate, a 1-unit odds’ increase in genetic liability to CAD was associated with reduced odds of mortality (OR 0.76; 95% CI 0.65, 0.90; *P* = 0.0018), directionally concordant with the individual level data analysis which was presented in terms of a 1 SD increase in CAD PRS (OR 0.91; 95% CI 0.85, 0.98) (see Table 1). After correction, the association between the CAD PRS and mortality increased in magnitude with a slightly more extreme inverse association, although confidence intervals overlap before and after correction (OR 0.72; 95% CI 0.60, 0.85; *P* = 0.0001).

## Discussion

In this study, we have demonstrated that the associations of CAD PRS with covariates and incident CV and fatal outcomes differ between those with and without prior CAD. Notably, we found that the associations of the PRS with a risk of future MI and CAD death were greatly attenuated among those with established CAD, with some evidence of a positive association for MI but very weak evidence for a positive association with CAD death, compared with those without CAD. Furthermore, we found evidence for inverse associations between the CAD PRS and all-cause death and ischaemic stroke amongst cases which were not present in individuals without known CAD.

These findings could be partially explained by index event bias, whereby stratifying on case status induces non-causal associations between genetic variants and risk factors for the index event. For example, individuals with high genetic risk for CAD may develop coronary disease despite low levels of conventional CAD risk factors such as smoking and adiposity. Indeed, we found some evidence that higher CAD PRSs are associated with a reduced BMI and smoking initiation in both samples, with more extreme effect sizes observed in the case sample suggesting that attenuated associations may be attributable to cases with higher genetic risk being otherwise healthier. Similarly, another possibility is that bias may be induced by the selection of prevalent CAD cases into UK Biobank; cases with high genetic risk for CAD may be more likely to die prior to being recruited into the study or decline participation for a health reason. This possibility is supported by the inverse association between the CAD PRS and age amongst cases, which suggests that cases with higher genetic risk for CAD may have an increased mortality. A further possibility is that the differences in associations are partially explained by aetiological heterogeneity between CAD onset and progression and characterised by differential drivers of stable and unstable plaque risk. However, it seems unlikely that the observed protective associations of the CAD PRS with all-cause death and ischemic stroke are explained by biological differences.

Medication use such as statins may also have contributed to the inverse associations in individuals with prevalent CAD, with previous evidence suggesting that statin use is more effective in those with higher genetic risk to CAD (19,21). This interaction likely relates to genetic overlap between CAD and LDL cholesterol, the target of statins, with higher genetic risk individuals more likely to have elevated LDL cholesterol. However, statins may be more effective in individuals with higher genetic risk that does not necessarily equate to lower absolute risk amongst individuals with elevated genetic risk, as our results imply for several outcomes. Indeed, in one of the previously cited studies (19), individuals with higher genetic risk were found to have increased mortality.

To investigate the potential effects of index event bias on our analyses, we applied two distinct methods. However, the two methods shifted the estimates in opposite directions; adjusting for covariates moved the estimates towards the non-case sample estimates, while the index event correction strengthened the inverse association between the CAD PRS and mortality amongst cases. One possible explanation for the increased inverse association after the index event correction is that the method assumes that the direct effects of prognosis and incidence are independent. In the context of coronary disease, there are clearly factors which influence both incidence and prognosis, such as LDL cholesterol, suggesting this assumption may not hold.

Our findings have important implications. First, although we did not formally evaluate prediction metrics, the modest odds ratios observed suggest that despite PRS positively associating with MI risk amongst diseased cases, existing PRSs are likely to have limited effectiveness for the prediction of subsequent events and therefore risk stratification in this setting (22). These findings imply that the genetic prediction of subsequent coronary disease events is likely to require dedicated GWAS of coronary disease progression. Second, our findings contribute to the existing literature (13,20,23–25) emphasising the caution required when using genetic data to infer causality in the context of disease progression. Genetic associations are generally thought to reflect causal effects because of the reduced possibility of confounding and reverse causation, but the observed protective associations of CAD PRS with mortality and ischemic stroke suggest that this may not hold for case-only studies. Index event bias has been shown to have modest impact on individual SNPs effects (23), but our results illustrate that bias likely accumulates when combining multiple markers together in a PRS, which could also affect Mendelian randomization studies.

Our study has notable limitations. First, our analyses used only the UK Biobank and require independent replication in different datasets and populations. Second, we could not differentiate between the effects of possible biases and genuine biological differences between onset and progression. Third, available biomarker data including LDL cholesterol were not available in UK Biobank at the time of writing, so we were unable to explore associations between the CAD PRS and CAD related biomarkers. Fourth, other researchers have derived more accurate PRS from the CardioGramPLUSC4D data than ours (5,6); however, individual risk prediction was not our goal, and given the positive association of our PRS with CAD incidence, we expect the same qualitative findings that would result from PRS including a greater number of weakly associated SNPs.

In conclusion, we have illustrated that associations between CAD genetic risk variants and CV outcomes differ when examined in those with and without prior CAD. This may be due to index event bias, although other possibilities need to be explored. Future work, such as dedicated GWAS of disease progression, by initiatives such as the GENIUS-CHD consortium (25) will aim to further explore genetic differences between the onset and progression of CAD.

## Materials and methods

### Data sources

#### UK Biobank

UK Biobank is a large-scale cohort study, which recruited approximately 500 000 individuals aged between 40 and 69 years from across the UK. Genotype data are available for the majority of participants with extensive phenotype data collected via questionnaire at baseline. Study participants are linked to electronic health record data from Hospital Episode Statistics (HES), secondary care data containing International Classification of Diseases, 10th Revision (ICD10) and Office of Population Censuses and Surveys Classification of Surgical Operations (OPCS) codes relating to study participants diagnoses and operative procedures. Study participants are also linked to the mortality register from the Office of National Statistics (ONS) which contains data on death, time of death as well as primary and secondary causes (26).

For the purposes of this study, we used a sample of 408 480 individuals, which was generated by starting with the full sample and removing individuals of non-European descent, individuals with more than 10 putative third-degree relatives in the kinship table and individuals who were flagged in quality control (sex mismatch, heterozygosity and individual missingness). We then defined two subsamples for our analyses: (a) baseline CAD controls, generated by removing prevalent CAD cases (see below) and individuals that self-reported as having had a heart attack (Field ID: 6150-0.0, 20002-0.0) or coronary angioplasty/coronary artery bypass grafts (Field ID: 20004-0.0) and (b) prevalent CAD cases identified using the following ICD10 (I21–I25, Z955) and OPCS codes (K40–K46, K471, K49, K50, K75) from HES occurring before their study enrolment date. In secondary analyses, CAD cases were stratified into CADMI cases (ICD10: I21–23, I241, I252) and CAD cases with no evidence of myocardial infarction (CADnoMI).

Phenotype data collected at baseline included sex, age, BMI (Field ID: 21001-0.0), SBP (Field ID: 4080-0.0), self-reported type II diabetes (Field ID: 2443-0.0), self-reported smoking status (Field ID: 20116-0.0) and self-reported statin use (Field ID: 20003).

Incident events after recruitment into the UK Biobank were ascertained using ICD10 and OPCS codes from HES using similar codes to published phenotyping algorithms (27). Incident CV events of interest included: MI (I21–23, I241, I252), heart failure (I110, I130, I132, I260, I50), ischemic stroke (I63, I693), stroke (I60–64, I69) and Revasc (K40–46, K471, K49, K50, K75). Fatal events of interest included CV death (CVD), CAD death and all-cause death and were ascertained using primary cause of death from mortality register data using ICD codes for cause-specific mortality. Composite events of interest were combined CAD death/MI and a combined variable including all CV outcomes. More information on incident outcomes and relevant ICD10 and OPCS codes is contained in Supplementary Material, Table S4.

UK Biobank study participants (*N* = 488 347) were assayed using the UK BiLEVE Axiom™ Array by Affymetrix1 (*N* = 49 950) and the closely related UK Biobank Axiom™ Array (*N* = 438 427). Directly genotyped variants were pre-phased using SHAPEIT3 (28) and imputed using Impute4 and the UK10K (29), Haplotype Reference Consortium (30) and 1000 Genomes Phase 3 (31) reference panels with post-imputation data including ~96 million genetic variants (32,26).

#### CARDIOGRAMPlusC4D

CARDIOGRAMPlusC4D (33) is a global collaboration of studies using a case-control approach to identify genetic variants associated with the presence of CAD. In this study, we used publicly available GWAS summary data from a recent consortium study independent of UK Biobank (2), which were downloaded from the CARDIOGRAM website (http://www.cardiogramplusc4d.org/data-downloads/).

### Statistical analysis

#### CAD PRS

We used GWAS summary data from CARDIOGRAMPlusC4D to construct a CAD PRS of SNPs. Initially, all SNPs meeting a *P*-value inclusion criterion (*P* < 5 × 10^−6^) were considered in order to generate a restrictive score containing only loci with relatively strong evidence for association with CAD. Highly correlated markers were then removed by LD clumping (*R*^2^ < 0.2, 250 kb distance threshold), the summary data using PLINK v1.9 (34) and the 1000 Genomes Phase 3 (GBR samples) (31). The final CAD PRS included 182 SNPs with the contribution of each SNP weighted by the GWAS effect estimates.

#### CAD PRS and incident events

We estimated associations between the CAD PRS and incident CV (stroke, ischemic stroke, myocardial infarction, heart failure and Revasc), fatal (all-cause death, CVD death and CAD death) and composite (all CVD, CAD death or MI) outcomes separately in the prevalent CAD case and baseline CAD free control samples. Logistic regression was used to estimate associations, with all analyses adjusted for age and sex. For comparison, we presented effect estimates in the two samples and tested for heterogeneity between these estimates (35). As a sensitivity analysis, we stratified the case only sample by the type of CAD (CADMI/CADnoMI) and compared estimates between the two samples with a test for heterogeneity. All estimates were presented in terms of the effect associated with a standard deviation increase in the PRS.

#### CAD PRS and baseline covariates

Index event bias may distort associations between different CAD risk factors (e.g. between CAD PRS and BMI) among cases potentially inducing correlations that are not present or are not as strong in samples of CAD free individuals. In turn, these may confound associations between risk factors and subsequent events (24). Therefore, we quantified and compared associations between CAD risk factors and the CAD PRS in the case and CAD-free samples.

As covariates of interest we chose established the risk factors for CAD available in UK Biobank (age, sex, SBP, BMI, type II diabetes, ever smoking and statin use), which were collected at study baseline. Linear or logistic regression models in R v3.6.0 were used to estimate the associations between the CAD PRS and covariates in the baseline CAD case and control samples. Analyses with age and sex, as the phenotypes of interest, were run unadjusted, with all other regression models including age and sex as covariates. For comparison, we presented the value of covariates of interest at quintiles (20, 40, 60 and 80%) of the CAD PRS distribution and formally tested for heterogeneity between estimates for first and subsequent events (35). We also evaluated the association between the CAD PRS and covariates in the whole unstratified UK Biobank sample (*N* = 408 480).

#### Accounting for index event bias

To evaluate the potential effects of index event bias on our analyses, we ran sensitivity analyses using two different approaches. First, we repeated the CAD PRS and incident events analyses in the baseline CAD case sample, including SBP, BMI, type II diabetes, ever smoking and statin use as covariates. These CAD risk factors were included as covariates to attempt to account for confounded associations between the CAD PRS and these covariates relating to index event bias.

Second, we used a recently proposed method to correct for index event bias in GWAS. SNP effects on prognosis (i.e. on events occurring after an index event) are adjusted using residuals from the regression of the SNP effects on the index event against the SNP effects on prognosis. The main caveat with the approach is that it assumes that the direct genetic effects on incidence and prognosis are independent (20).

In this instance, the index event is existing CAD, so we used GWAS summary data from the CARDIOGRAMPlusC4D GWAS (2). For a GWAS of prognosis, we used the UK Biobank CAD case sample (*N* = 10 287) to perform a GWAS of all-cause mortality using a logistic model in snptest v2.5.2 (36), including age, sex and the first 10 principal components as covariates. As suggested previously in Dudbridge *et al.* which outlined the IndexEvent adjustment (20), we then extracted 116 438 independent SNPs common to both the CARDIOGRAMPlusC4D and the UK Biobank GWAS summary statistics by restricting to well-imputed SNPs (INFO > 0.99) and LD pruning (250 kb step window, 5 SNP step size, *r*^2^ = 0.1) using the 1000 Genomes GBR samples (Phase 3) (31) as a reference panel. These SNPs were then used to calculate the slope correction estimate using the SIMEX (37) option in the IndexEvent.R package with a Hedges-Olkin estimate calculated as a sensitivity analysis. When applying this in practice, the SIMEX slope estimates did not converge using all SNPs, even with 10 000 simulations, so we decided to reduce noise by removing SNPs which were not strongly associated with the index trait and calculate the slope using a subset of 5564 SNPs which were nominally associated with the index trait in CARDIOGRAMPlusC4D (*P* < 0.05). We calculated the *I*^2^ G_X_ statistic for this subset of 5564 SNPs using formulae contained in a previous publication (37), to estimate the degree of measurement error which could lead to attenuation in SIMEX estimates.

Next, we adjusted the betas and standard errors in the UK Biobank GWAS of mortality using the slope of the regression. For example, the adjusted betas were calculated by subtracting the product of the slope estimate and the CARDIOGRAM incidence beta estimate from the prognosis beta for each SNP. Finally, to estimate the association between the CAD PRS and mortality among CAD cases from summary data (instead of individual level data as previously), we used an inverse-variance weighted method (38,39) across 54 independent SNPs (33) using CAD as the exposure and mortality as the outcome. The subset of chosen independent SNPs reached genome-wide significance in the largest GWAS independent of UK Biobank. Estimates were presented in terms of the association of an increase in the CAD PRS, corresponding to an odds increase of CAD, with log-odds of mortality. For comparison, we estimated the PRS association before and after correction.

## Data Availability

The summary statistics for the GWAS of all-cause mortality amongst CAD cases in UK Biobank will be uploaded to the GWAS catalog.

## Funding

British Heart Foundation Intermediate Fellowship (Dr Patel, grant number FS/14/76/30933); National Institute for Health Research University College London Hospitals Biomedical Research Centre; British Heart Foundation grant number PG/18/5033837 to A.F.S.; University College London Hospitals National Institute for Health Research Biomedical Research Centre, European Union/European Federation of Pharmaceutical Industries and Associations Innovative Medicines Initiative 2 Joint Undertaking BigData@Heart grant no 116074 to F.W.A.; European Union’s Horizon 2020 research and innovation programme under the ERA-NET Co-fund action No. 01KL1802 (Druggable-MI-gene); Dutch Heart Foundation and Netherlands Organization for Health Research and Development (ZonMw). Professor Hingorani is a National Institute for Health Research Senior Investigator. The funder(s) of the study had no role in study design, data collection, data analysis, data interpretation or writing of the report.

## Conflicts of Interest Statement

Dr Patel has received speaker fees and honoraria from Amgen, Sanofi and Bayer and research grant funding from Regeneron. Dr Asselbergs has received research funding from Regeneron, Pfizer and Sanofi. The other authors report no conflicts.

## Supplementary Material

Supplementary_12_03_20_ddaa052
