# Supplementary material for: Polygenic risk scores for coronary artery disease and subsequent event risk amongst established cases
Source: Hum Mol Genet. 2020 Mar 27;29(8):1388–95. doi: 10.1093/hmg/ddaa052 (PMC7254844; doi:10.1093/hmg/ddaa052)
Supplement: Supplementary_12_03_20_ddaa052 [file supplementary_12_03_20_ddaa052.docx]

**Supplementary Table 1** CAD subtypes and incident events

| **Outcome** | **Prevalent CADMI cases (N=3,881)**  OR (95% C.I.) | **Prevalent CAD/NOMI cases (N=6,406)**  OR (95% C.I.) |
| --- | --- | --- |
| CAD death/MI | 1.09 (0.97, 1.21) | 1.12 (1.01, 1.23) |
| CV death | 0.96 (0.80, 1.15) | 0.95 (0.82, 1.10) |
| All-cause death | 0.91 (0.81, 1.01) | 0.91 (0.83, 0.99) |
| CAD death | 0.92 (0.76, 1.13) | 0.95 (0.81, 1.11) |
| MI | 1.11 (0.98, 1.27) | 1.17 (1.05, 1.31) |
| Revasc | 0.97 (0.84, 1.13) | 0.90 (0.81, 1.00) |
| Heart failure | 0.99 (0.80, 1.21) | 0.99 (0.84, 1.15) |
| Stroke | 0.98 (0.80, 1.19) | 0.82 (0.70, 0.96) |
| Ischaemic stroke | 0.87 (0.69, 1.10) | 0.72 (0.60, 0.87) |
| All CVD | 1.03 (0.94, 1.12) | 0.96 (0.89, 1.03) |

^1 All OR per 1 S.D. increase in CAD GRS of 182 SNPs^

**Supplementary Table 2** Association of CAD GRS with baseline covariates in the whole UK Biobank sample

| **Covariate** | **Whole UK Biobank sample (N=408,840)**  Change in covariate per 1 S.D. increase in CAD PRS of 182 SNPs (95% C.I.) |
| --- | --- |
| Age (Years) | -0.007 (-0.031, 0.018) |
| Sex  (Male=1  Female=0) | OR 1.00 (0.99, 1.00) |
| Statin use  (Yes=1  No=0) | OR 1.20 (1.18, 1.21) |
| Type II Diabetes  (Yes=1  No=0) | OR 1.04 (1.02, 1.06) |
| SBP (mmHg) | 0.38 (0.32, 0.44) |
| BMI (kg/m2) | -0.03 (-0.04, -0.01) |
| Smoking (Ever=1  Never=0) | OR 1.00 (0.99, 1.01) |

**Supplementary Table 3** Association of CAD PRS with subsequent incident events adjusted for covariates

| **Outcome** | **Prevalent CAD cases (N=10,287)**  OR (95% C.I.) | |
| --- | --- | --- |
|  | **Adjusted for age and sex.** | **Adjusted for age, sex, statin-use, SBP, diabetes, BMI and ever smoking.** |
| CAD death/MI | 1.11 (1.03, 1.20) | 1.14 (1.06, 1.24) |
| CV death | 0.97 (0.87, 1.09) | 0.98 (0.87, 1.11) |
| All-cause death | 0.91 (0.85, 0.98) | 0.92 (0.85, 0.99) |
| CAD death | 0.96 (0.85, 1.08) | 0.98 (0.86, 1.12) |
| MI | 1.15 (1.06, 1.25) | 1.18 (1.08, 1.29) |
| Revasc | 0.91 (0.84, 1.00) | 0.90 (0.82, 0.98) |
| Heart failure | 0.97 (0.86, 1.10) | 1.04 (0.91, 1.19) |
| Stroke | 0.88 (0.78, 1.00) | 0.92 (0.81, 1.05) |
| Ischaemic stroke | 0.78 (0.67, 0.90) | 0.82 (0.70, 0.95) |
| All CVD | 0.99 (0.93, 1.04) | 1.00 (0.94, 1.06) |

^1 All OR per 1 S.D. increase in CAD PRS of 182 SNPs^

**Supplementary Table 4** Codes used to phenotype cardiovascular and fatal endpoints

| **Endpoint** | **Data source** | **Codes** | **Number of incident events (%)** | |
| --- | --- | --- | --- | --- |
|  |  |  | **No CAD at baseline (N=393,108)** | **Prevalent CAD cases (N=10,287)** |
| MI | Hospital episodes statistics | I21-23*, I241, I252 | 2,848 (0.7%) | 575 (5.6%) |
|  | Mortality: Primary cause of death | I21-23*, I241, I252 |  |  |
| Stroke | Hospital episodes statistics | I60-64*, I69* | 2,699 (0.7%) | 273 (2.6%) |
|  | Mortality: Primary cause of death | I60-64*, I69* |  |  |
| Heart failure | Hospital episodes statistics | I110, I130, I132, I260, I50* | 723 (0.2%) | 258 (2.5%) |
|  | Mortality: Primary cause of death | I110, I130, I132, I260, I50* |  |  |
| Ischaemic stroke | Hospital episodes statistics | I63*, I693 | 1,690 (0.4%) | 194 (1.9%) |
|  | Mortality: Primary cause of death | I63*, I693 |  |  |
| Revascularization | OPCS codes | K40-46, K471, K49, K50, K75 | 11,654 (3.0%) | 553 (5.4%) |
| All-cause death | Mortality | Any fatal event | 10,516 (2.7%) | 865 (8.4%) |
| CAD death | Mortality: Primary cause of death | I20-25*, R96 | 921 (0.2%) | 264 (2.6%) |
| CVD death | Mortality: Primary cause of death | I20-25*, R96, I46*, I49*, I110, I130, I132, I260, I50*, I60-64*, I69*, I171*, I173* | 1,441 (0.4%) | 313 (3.0%) |
| CAD death/MI | Composite of MI/CAD death |  | 3,359 (0.9%) | 729 (7.1%) |
| All CVD | Composite of CVD death, MI, stroke and revascularization |  | 17,531 (4.5%) | 1,487 (14.5%) |

**Supplementary Figure 1** Mean age across quintiles of the CAD GRS distribution in prevalent CAD cases and controls.

**
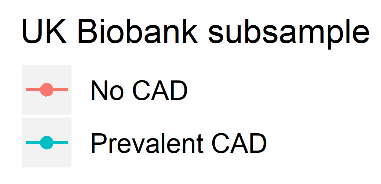
*
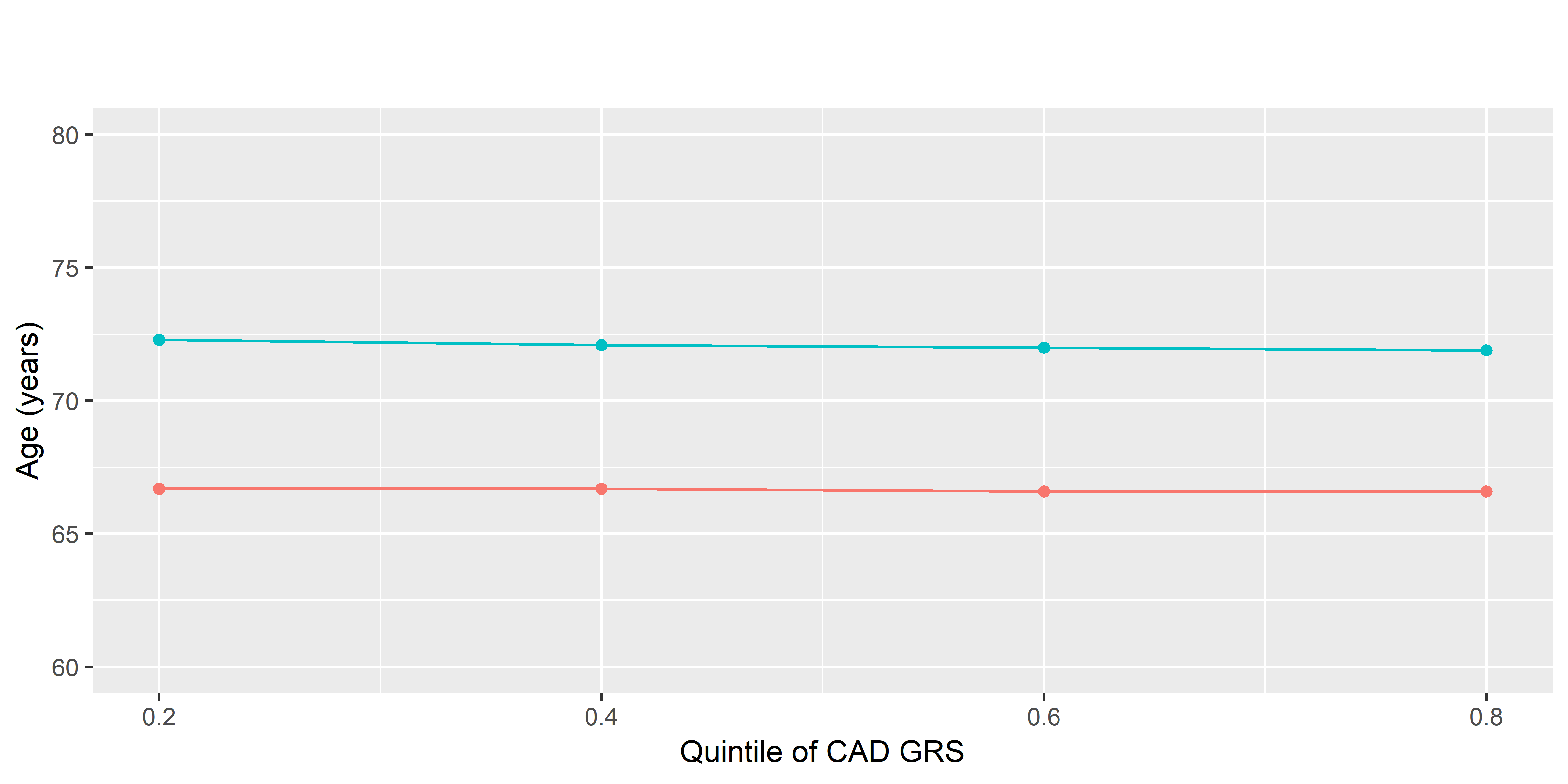
***

**Supplementary Figure 2** Sex distribution across quintiles of the CAD GRS distribution in prevalent CAD cases and controls.

**
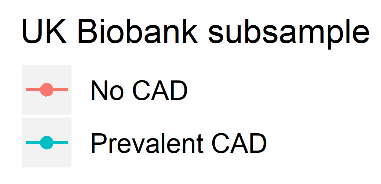
**
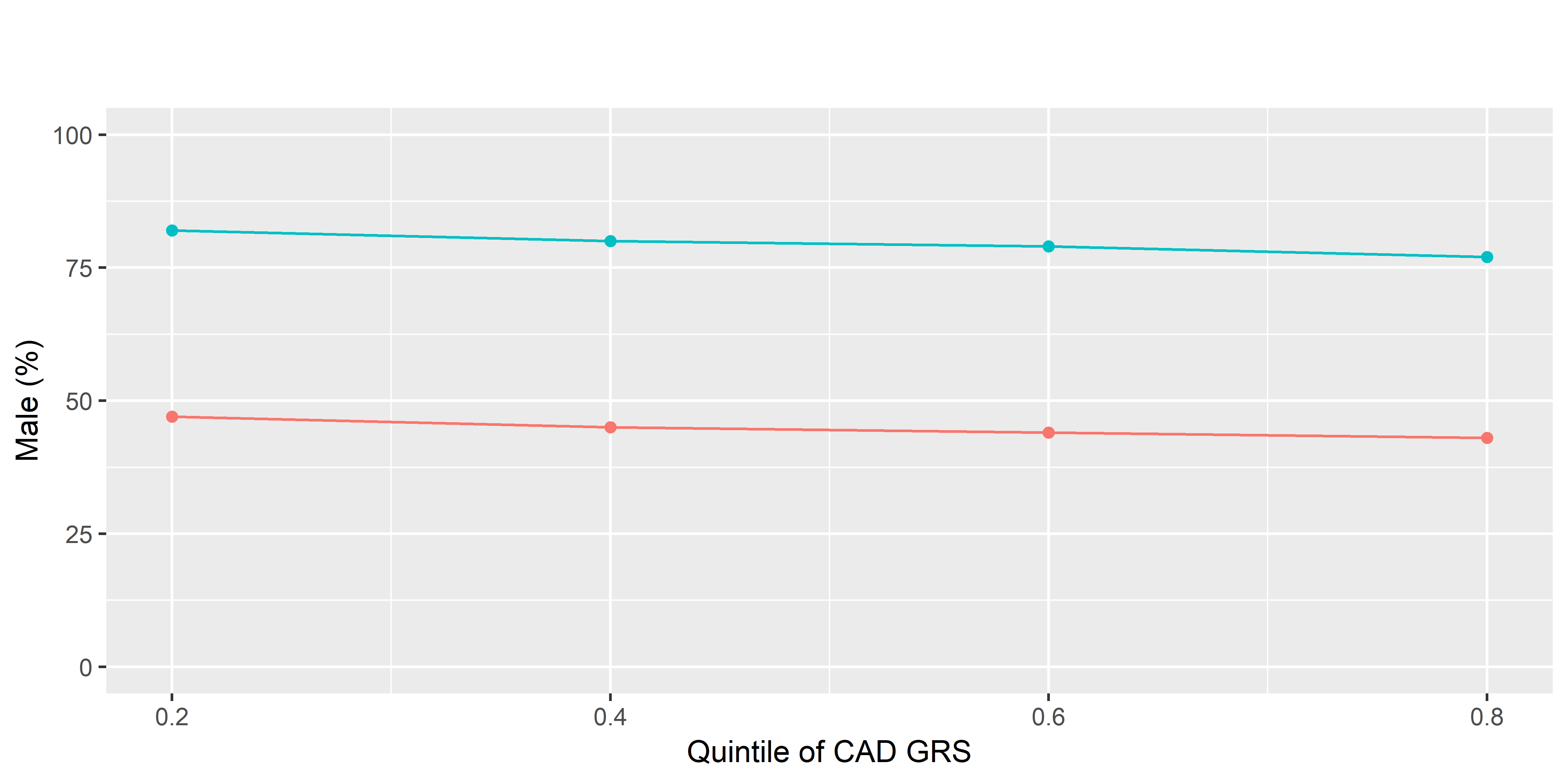


**Supplementary Figure 3** Statin use distribution across quintiles of the CAD GRS distribution in prevalent CAD cases and controls.

**
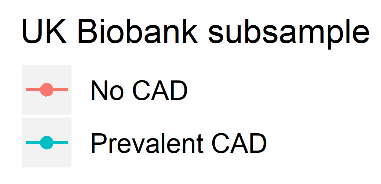

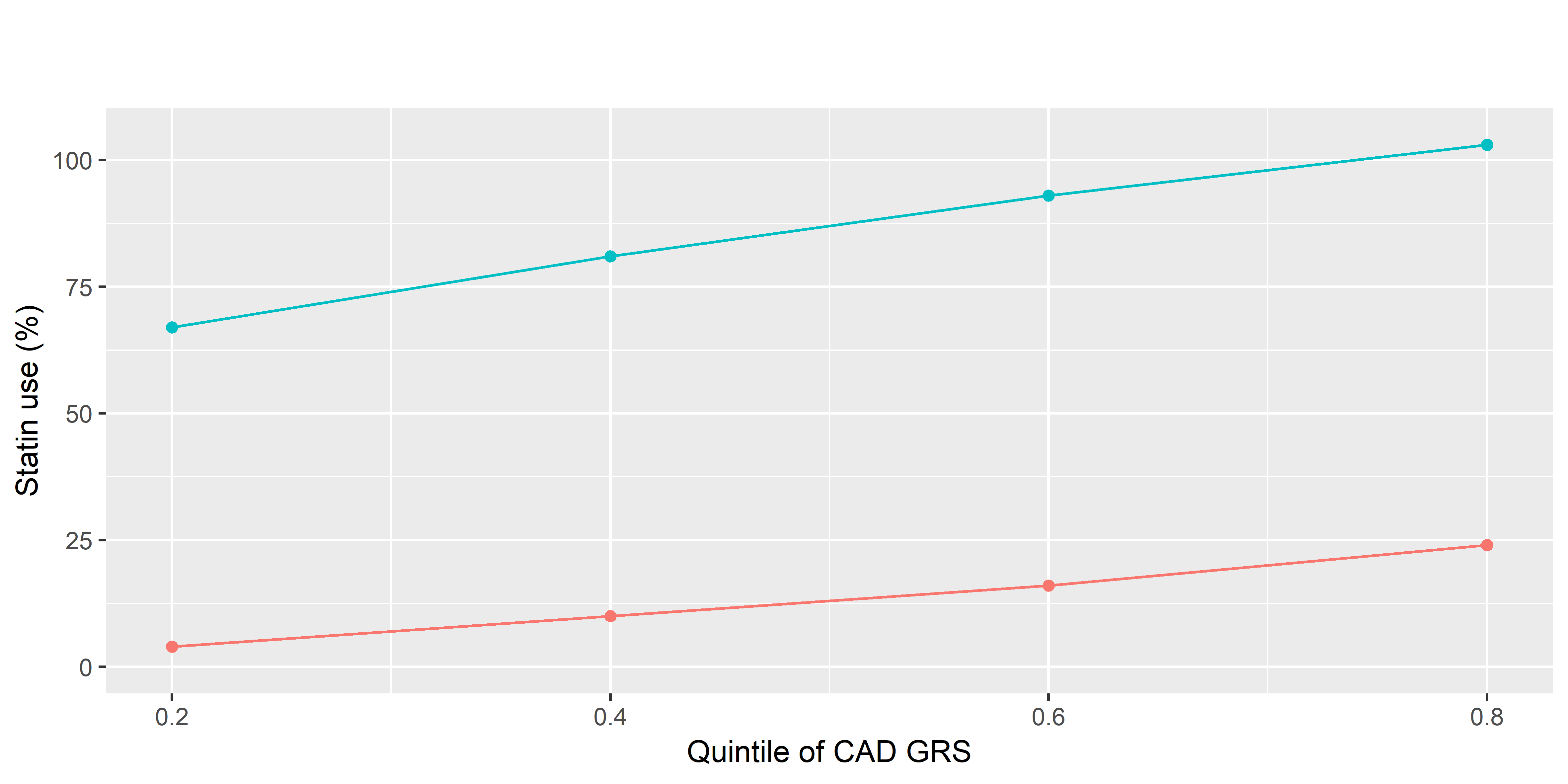
**

**Supplementary Figure 4** Prevalence of Type 2 Diabetes across quintiles of the CAD GRS distribution in prevalent CAD cases and controls.

**
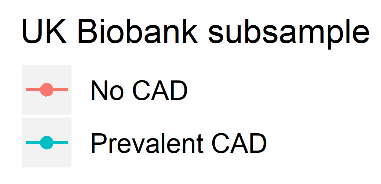

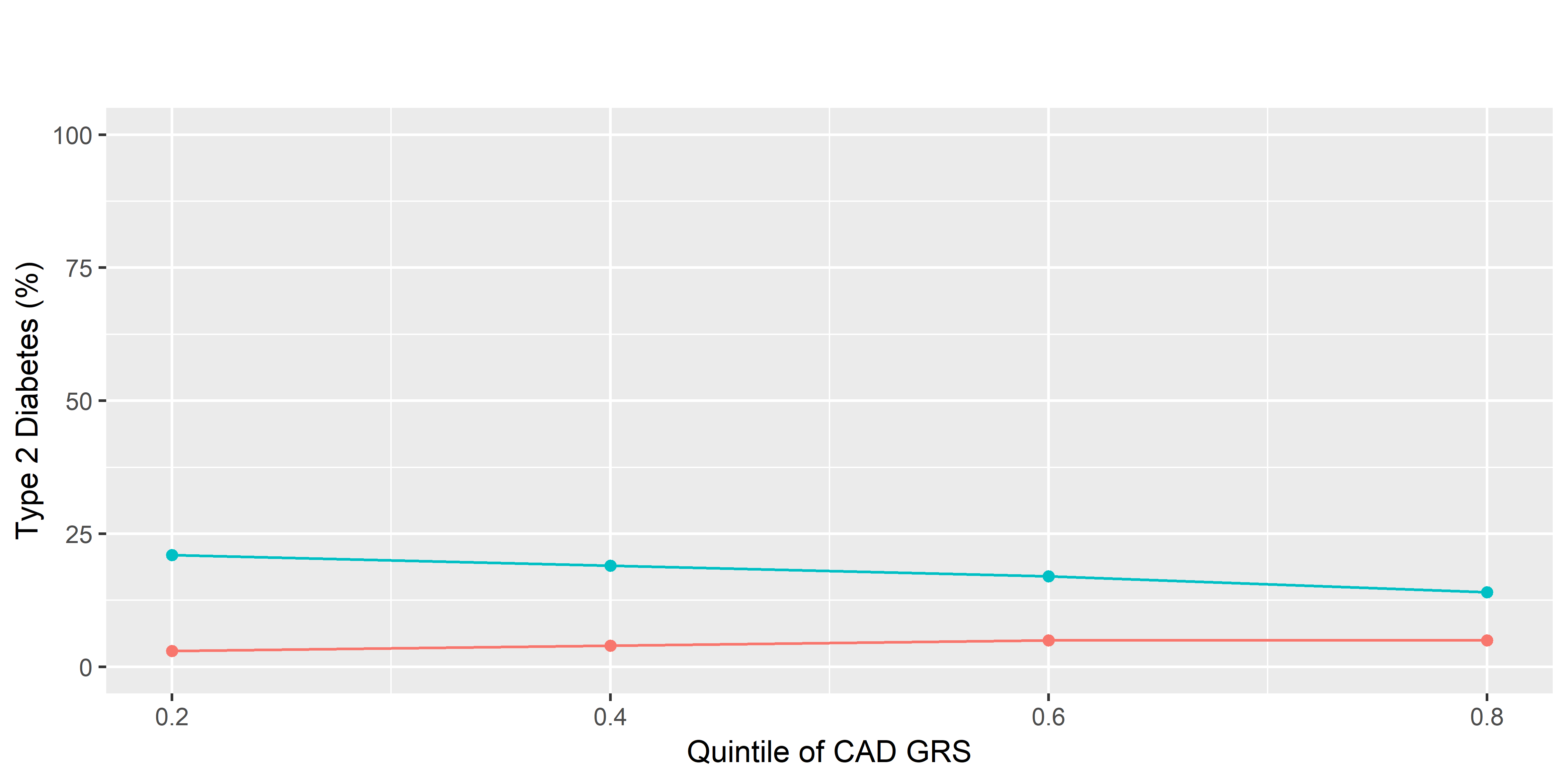
**

**Supplementary Figure 5** Systolic blood pressure across quintiles of the CAD GRS distribution in prevalent CAD cases and controls.

**
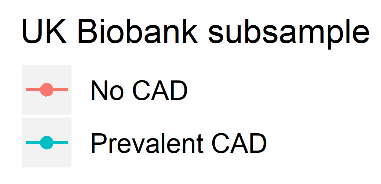

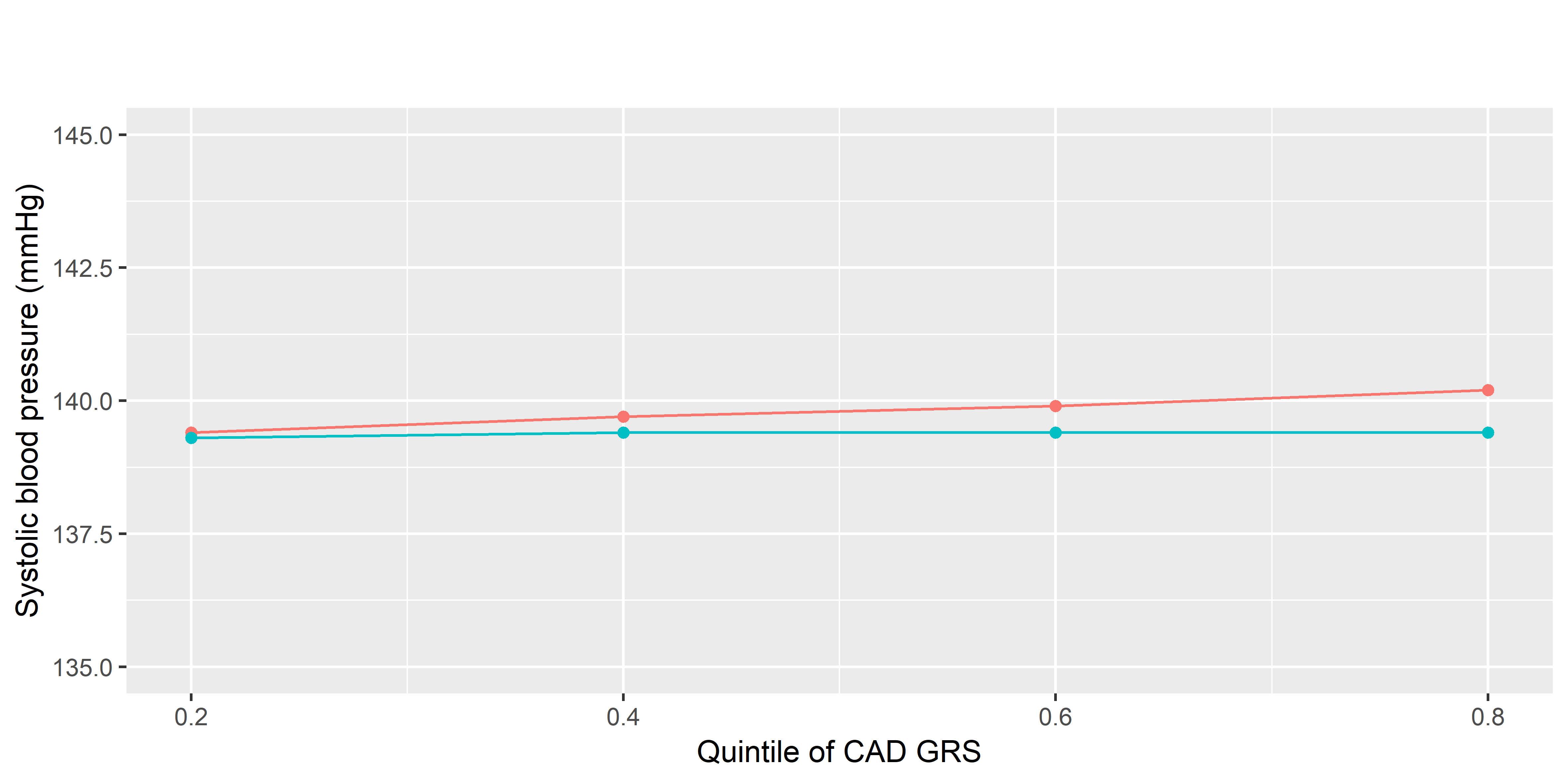
**

**Supplementary Figure 6** BMI across quintiles of the CAD GRS distribution in prevalent CAD cases and controls.

**
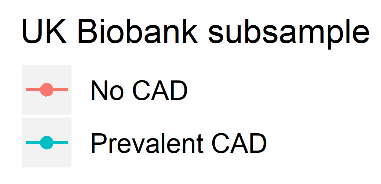

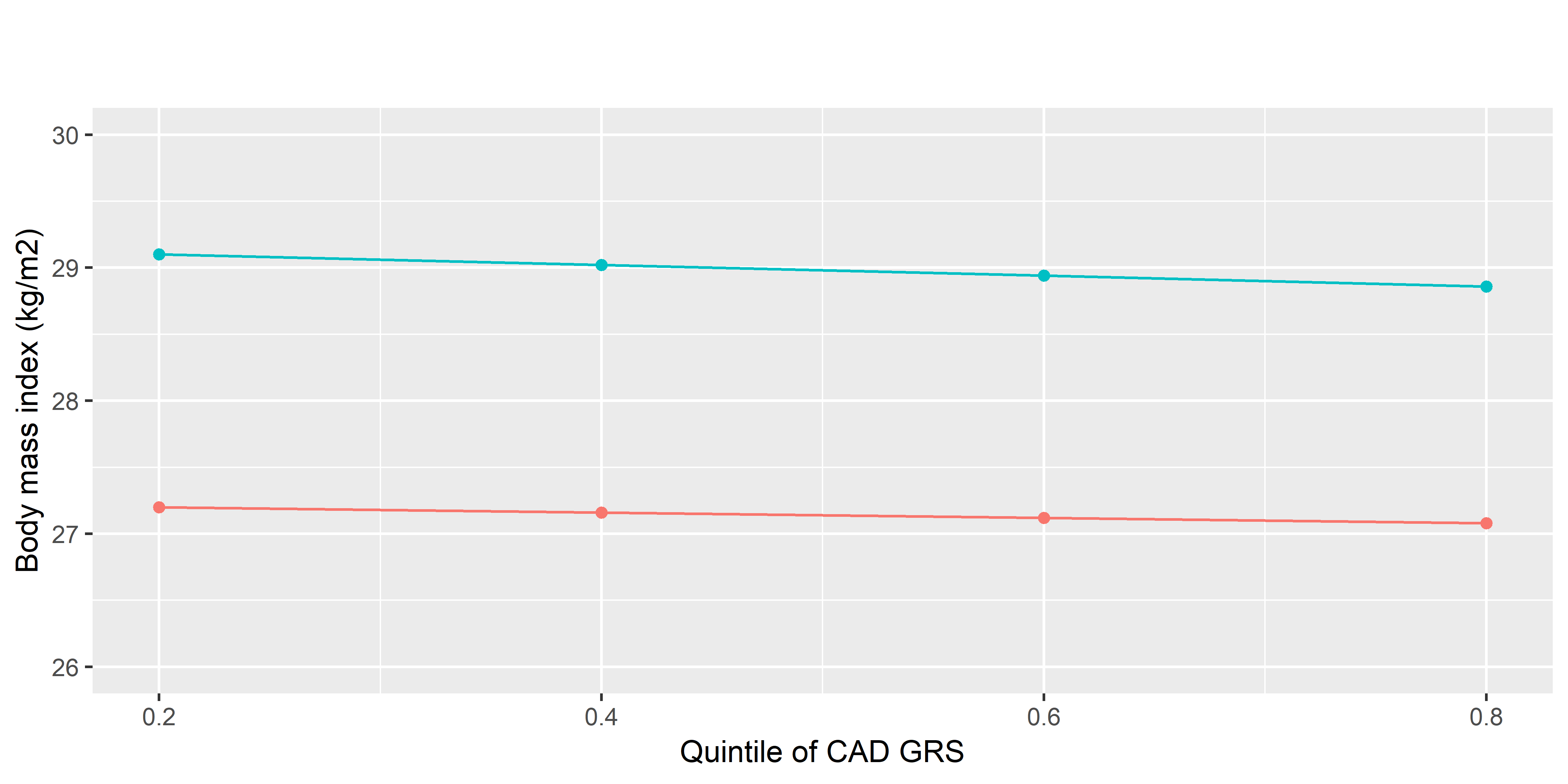
**

**Supplementary Figure 7** Prevalence of ever smoking across quintiles of the CAD GRS distribution in prevalent CAD cases and controls.

**
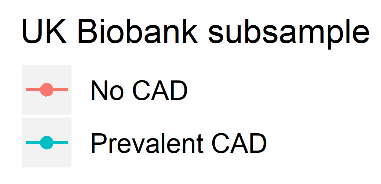

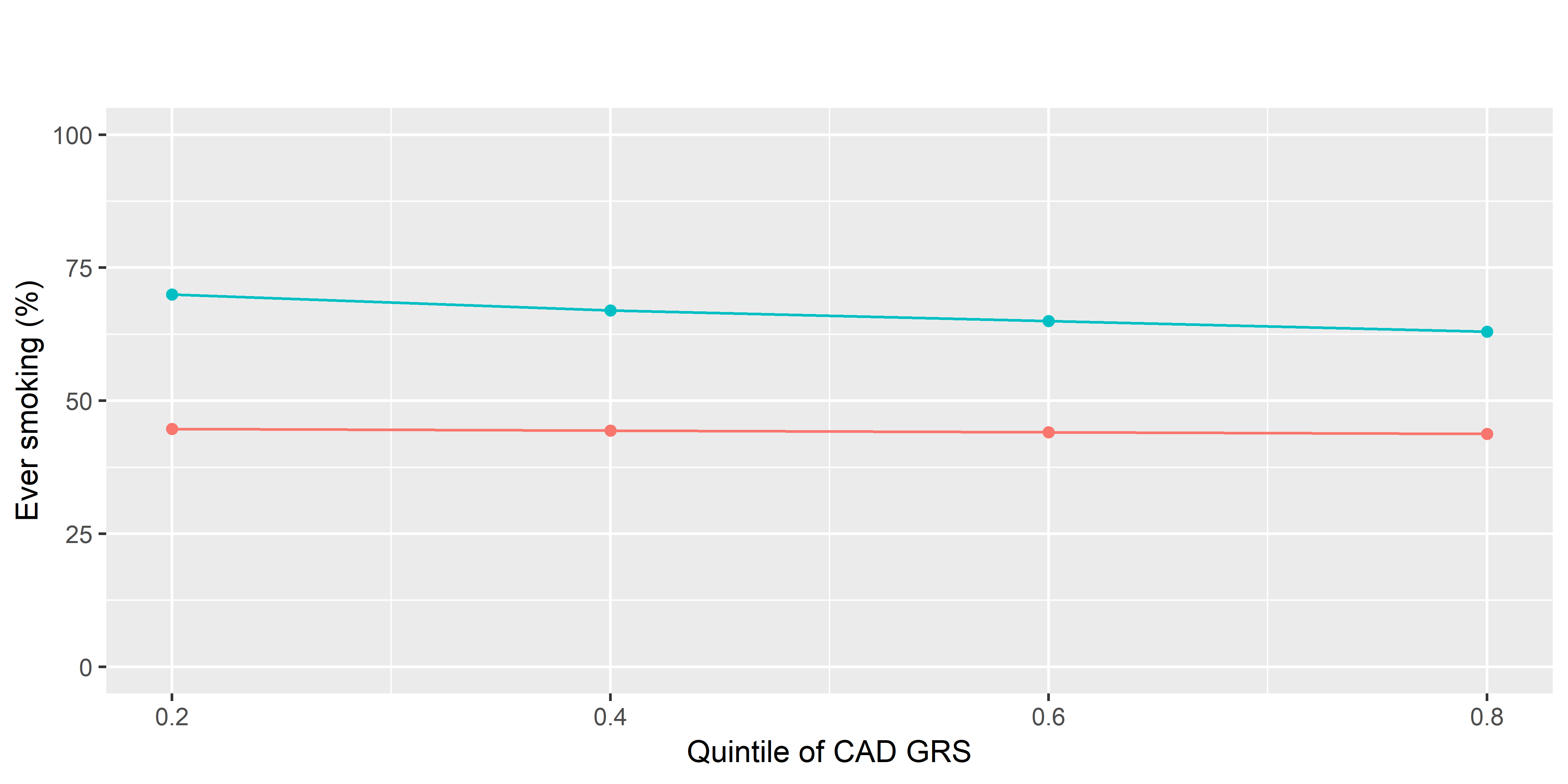
**
